# A Retrospective Analysis: Autologous Peripheral Blood Hematopoietic Stem Cell Transplant Combined With Adoptive T-Cell Therapy for the Treatment of High-Grade B-Cell Lymphoma in Ten Dogs

**DOI:** 10.3389/fvets.2021.787373

**Published:** 2021-12-07

**Authors:** Alexandra Gareau, Alexandra Z. Ripoll, Steven E. Suter

**Affiliations:** ^1^Department of Clinical Sciences, North Carolina State University College of Veterinary Medicine, Raleigh, NC, United States; ^2^North Carolina State University Comparative Medicine Institute, Raleigh, NC, United States; ^3^Duke/NCSU Consortium for Comparative Canine Oncology, Raleigh, NC, United States

**Keywords:** adoptive T-Cell therapy, dogs, lymphoma, hematopoietic stem cell transplantation, CHOP chemotherapy

## Abstract

In humans, a type of cellular immunotherapy, called adoptive T cell transfer (ACT), can elicit curative responses against hematological malignancies and melanoma. ACT using *ex vivo* expanded peripheral blood T-cells after multiagent chemotherapy enhances tumor-free survival of dogs with B-cell lymphoma (LSA). Since 2008, our group has been performing autologous peripheral blood hematopoietic stem cell transplants (autoPBHSCT) for the treatment of canine high-grade B-cell LSA, although relapse of residual disease is a common cause of reduced survival in ~70% of treated dogs. We reasoned that a more aggressive treatment protocol combining CHOP (cyclophosphamide, doxorubicin, vincristine, prednisone) chemotherapy, autoPBHSCT, and ACT to treat 10 dogs with B-cell LSA could lead to better outcomes when compared to dogs treated with CHOP chemotherapy and autoPBHSCT alone. Using this protocol, once dogs achieved complete hematologic reconstitution post-autoPBHSCT, CD3+ CD8+ and CD3+CD4+ T-cells were expanded from the peripheral blood at a commercial laboratory. Two to four ACT infusions were given to each dog, with a total of 23 infusions given. Infusions were administered with no complications or adverse events. The median cell dose for all infusions was 5.62 x 10^6^ cells/kg (range: 2.59 x 10^6^-8.55 x 10^6^ cells/kg). 4/10 (40%) of dogs were cured of their disease (defined as disease-free for ≥2 years post-autoPBHSCT). Our results confirm that the autoPBHSCT protocol did not hinder the *in vitro* expansion of autologous peripheral blood T-cells and that the final product could be administered safely, with no adverse events recorded. Finally, since only ten dogs were treated, our results can only suggest that the administration of ACT to dogs after multiagent chemotherapy and autoPHSCT did not lead to a statistically significant increase in median disease-free interval and overall survival when compared to dogs who received CHOP chemotherapy and autoPHSCT alone.

## Introduction

Autologous peripheral blood hematopoietic stem cell transplantation (autoPBHSCT) is an aggressive treatment option for dogs with hematologic malignancies (1–3). AutoPBHSCT restores complete hematologic reconstitution by infusion of autologous CD34+ hematopoietic stem cells after intensive myeloablative therapy. When used in conjunction with CHOP-based multiagent chemotherapy, autoPBHSCT achieves a cure (as defined as being disease-free > 2 years post autoPBHSCT) in 30% and 19% of dogs with B- and T-cell LSA, respectively (1, 2). Regardless of whether chemotherapy ablation or radiation ablation is utilized, relapse of residual disease, leading to reduced survivals, is common in people (4) and dogs due to the inability of either ablative therapy to kill every malignant lymphocyte in the body of both species.

In an effort to increase malignant lymphocyte cell killing, an allogeneic peripheral blood hematopoietic stem cell transplant (alloPBHCST), using DLA-matched donor stem cells, can be considered. In this setting, cell killing is achieved due to the effects of graft vs. host disease (GVHD) and subsequent graft vs. tumor (GVT) effects (5, 6), rather than the direct killing using radiation ablation. GVT is mediated through allogeneic activated T-lymphocytes that attack residual cancer cells by recognizing and responding to recipient tumor antigens, which leads to better outcomes (7–11). As such, while not utilized in this study, alloPBHSCT represents one of the earliest forms of immunotherapy that emphasizes the utility of T-cells as very effective immunotherapy agents.

Adoptive T-cell therapy (ACT) utilizes the isolation, *ex vivo* activation, and infusion of autologous antigen-specific (i.e., CAR T-cells) or non-specific lymphocytes (i.e., TILs, PBLs) that have the potential to enhance cell-mediated antitumor immunity (12). This strategy aims to eliminate residual cancer cells through direct anti-neoplastic effects, or through indirect effects mediated by immunity directed against elements supporting tumor growth (13). In veterinary medicine, although CD20-targeted CAR T-cells have been manufactured and safely infused into dogs with high-grade B-cell LSA (14), CAR T-cell products are not currently available for routine clinical use. To the author's knowledge, there are only two reports in the veterinary literature documenting the use of ACT to treat canine malignancies. The first is the treatment of dogs with appendicular osteosarcoma consisting of *in vitro* tumor cell expansion, intradermal tumor cell injections, mononuclear cell collection and *in vitro* expansion, and infusion of expanded CD3+ T-cells followed by serial IL-2 injections (15). The second is the use of *in vitro* expanded autologous peripheral blood CD3+ T-cells to treat dogs with high-grade B-cell LSA as an adjunctive treatment following CHOP chemotherapy (16). Using this approach, peripheral blood mononuclear cells were propagated for 28–35 days on γ-irradiated OKT3-loaded aAPC (human K562 erythroleukemic cell line transduced with lentiviruses to co-express human CD19, CD46, CD86, and CD137L, and membrane bound hIL-15) with rhIL-2/IL-12, to expand a population of T-cells composed primarily of CD3+CD8+ T-cells (54 ± 6%) and CD3+CD4+ T-cells (33 ± 3%). These cells were safely infused into eight dogs with B-cell LSA, seven of which were in clinical remission (CR) after receiving a standard course of CHOP chemotherapy. When compared to a historical control population of 12 dogs achieving a CR after CHOP chemotherapy, the dogs who received ACT had a statistically significant improvement in median overall survival (OS, 392 vs. 167 days) and median disease-free survival (DFI, 338 vs. 71 days).

Expansion of canine peripheral blood CD3+ T-cells for use as ACT in the treatment of dogs with B-cell LSA is currently available from two commercial US laboratories, although there are no reports in the literature, to our knowledge, that document their safety or efficacy in a clinical setting. Importantly, there are also no manuscripts in the literature describing the cellular content of the final infused products. Based on the promising aforementioned data (16), and since both autoPBHSCT and ACT costs are covered by most pet insurance companies in the US, we reasoned that adding ACT to our autoPBHSCT protocol could improve the clinical outcome of dogs when compared to autoPBHSCT alone. In this retrospective study, we describe the use of autoPBHSCT and ACT to treat ten dogs with B-cell LSA after CHOP chemotherapy to (1) assess the feasibility of using the product and the safety of multiple infusions, and, (2) determine whether there was a suggestion that ACT improved their overall clinical outcomes when compared to a historical group of dogs treated with CHOP chemotherapy and autoPBHSCT alone.

## Materials and Methods

### Autologous PBHSCT

The medical record database at North Carolina State University Veterinary Hospital was searched for dogs with a diagnosis of high-grade B-cell LSA who received both autoPBHSCT and ACT. Ten dogs who received an autoPBHSCT as previously described (1, 2) and ACT, after receiving CHOP chemotherapy to induce clinical remission, were identified. Briefly, all dogs received high dose cyclophosphamide (500–650 mg/m2) administered 2 weeks prior to presentation for autoPBHSCT. After complete staging, mobilization was performed for 5 days using Neupogen (rhG-CSF, 5 ug/kg SC BID). After a double dose of Neupogen on the morning of d5, peripheral blood mononuclear cells were collected with either a COBESpectra^®^ or Terumo Optia^®^ apheresis system using a continuous mononuclear cell collection protocol, as previously described (17, 18).

### Apheresis Product Analysis

Apheresis products underwent polymerase chain reaction for antigen receptor rearrangements (PARR) if the tumor was originally PARR positive (19). Quantification of CD34+ cells in the apheresis products was previously described (1, 2). All harvest products were refrigerated at 4°C without any manipulation until transplantation 2 days later.

### Total Body Irradiation

All dogs received total body irradiation (TBI) consisting of either 10 Gy divided into two 5 GY fractions given on two successive days (5 dogs) or 12 Gy divided into two 6 Gy fractions given on two successive days (5 dogs) at a dose rate of 8.5 cGy/min administered with 6 MV photons from a Varian Clinac 1,800, as previously described (20). The increase in total TBI dose from 10 to 12 Gy was made in an effort to increase the neoplastic cell killing efficacy of TBI, in light of the ~70% relapse rate of dogs with B-cell LSA treated with chemotherapy and autoPBHCST alone.

### Post-autoPBHSCT Care

Immediately after TBI, the apheresis product was warmed to room temperature and administered intravenously (IV) over a period of 30 min. Post-autoPBHSCT care in the hospital was essentially as previously described (1, 2). All dogs achieved complete hematologic reconstitution as previously described (21), although no dogs had platelet counts >100 k/uL when discharged from the hospital. Follow-up care after discharge included weekly recheck visits at the referring veterinarian for physical examinations and complete blood counts (CBC) until platelets were >100,000/uL. Thereafter, monthly physical examinations were performed for 6 months. After 6 months, the clinical status of the dogs was determined via regular communication with the owners and/or referring veterinarians.

### Propagation of T-Cells

Upon evidence of complete hematologic reconstitution (WBC ≥ 4.4 k/uL, neutrophils ≥ 2.8 k/uL, PLTs ≥ 100 k/uL), 20 mL of peripheral blood was collected into EDTA tubes and shipped chilled overnight to a commercial laboratory (Aurelius Biotherapeutics©, https://aureliusbio.com/) for CD3+ T-cell expansion using *in vitro* techniques similar to those previously described (16), with proprietary modifications to increase cell yield and decrease shipping related cell death. This laboratory is in compliance with Veterinary Services Memorandum (VSM) No. 800.50 to manufacture the product using a controlled, consistent process, and VSM 800.121 of the USDA Center for Veterinary Biologics (CVB) to ship autologous therapeutic biologics. USDA licensure of the product is pending. As described previously (16), the total cell count of the *in vitro* propagated cells dropped significantly during the first week of expansion and then expanded in subsequent weeks (data not show).

### Characterization of Propagated T-Cells Used for ACT

Although the final number of expanded T-cells was reported with their product, the immunophenoyptic characterization of the ACT products produced at Aurelius Biotherapeutics^©^ used in this study were not determined. However, flow cytometric analysis (Hematologics, Inc. [www.hematologics.com] and Colorado State University Clinical Immunology Laboratory) of 16 randomly chosen ACT samples supplied to us by the company confirmed the variable expansion of a population of small, viable, 7-AAD-/CD45+/CD5+ lymphocytes comprised of varying CD4:CD8 ratios, admixed with a variable number of 7AAD +/CD5-/CD45- cells (non-viable cells and cellular debris). In 4 samples, a population of viable 7+AAD-/CD45-/CD5-/CD4-/CD81+ NK cells were also seen. In brief, cells were first stained with 7-AAD to identify live cells, and then sequentially stained with CD45 (to identify leukocytes), CD5 (to identify T-cells) and CD4/CD8 (to determine CD4+/CD8+ ratio). A summary of this data is provided in Supplementary Table 1, and a representative flow cytometric staining strategy of an ACT product (analyzed by Hematologics, Inc.) is shown in Supplementary Figure 1.

### Infusion of Propagated ACT Product

After ~2 weeks of *in vitro* expansion, the cells were harvested, collected into a sterile custom manufactured BioLoc^TM^ needle-free cell transfer bag (Instant Systems, Inc., Norfolk, VA, US, Supplementary Figure 2) containing ~20 ml of activated T-cells and sent chilled for overnight delivery to referral practices. After warming to room temperature, the T-cells were infused IV over 30 min at varying intervals per the manufacturers' suggested protocol (Supplementary Figure 3). Some dogs received more infusions, which was dependent on the efficacy of the *in vitro* expansion. All dogs were premedicated with diphenhydramine (1 mg/kg) and maropitant (1 mg/lb) prior to infusion and were monitored for immediate and delayed transfusion reactions.

### Statistical Analysis

Disease-free interval (DFI), defined as the time from autoPBHCST until the time of relapse, and overall survival (OS), defined as the time from autoPBHCST until death, were calculated using the Kaplan–Meier method. DFI and OS were compared between these dogs and a historical control group treated with autoPBHSCT only (1) using the log rank test. There was no statistically significant difference between these 2 groups of dogs when comparing mean age (5 vs. 6.7 years), mean weight (31 vs. 33kgs), or mean days from diagnosis to autoPBHSCT (94 vs. 150 d) In addition, when these two groups were combined (34 total dogs), 97% of the dogs were diagnosed with Stage III-Va lymphoma. For the historical control group, three dogs (two who died in the hospital six and 10 days post-PBHSCT and one who died of apparent graft failure 45 days post-PBHSCT) were censored from the disease-free analysis. One additional dog who was lost to follow up was censored from the overall survival analysis. All statistics were calculated using GraphPad Prism 8 (version 8.4.3). A *p*-value ≤ 0.05 was considered significant.

## Results

### Patient Characteristics

Ten dogs, representing 6 breeds, diagnosed with high-grade B-cell LSA presented to the North Carolina State Veterinary Hospital Bone Marrow Transplant Unit. (Table 1). The mean age was 5 years old (range: 3–6 years old). Seven dogs were diagnosed via enlarged lymph nodes (LN) fine needle aspirates and three dogs were diagnosed via histopathology of LN biopsies. Staging was performed according to the World Health Organization staging system for dogs with LSA. At diagnosis, five dogs had stage III disease, two had stage IV disease and three had stage V disease. Immunophenotype assignment was determined using PARR on LN cytologic samples (6 dogs), flow cytometry of LN FNAs (2 dogs), or immunohistochemistry of formalin-fixed paraffin embedded LN tissue (2 dogs). Eight dogs had B-cell PARR positive tumors, one dog was B-cell PARR negative and one dog did not have PARR run due to a lack of available tissue. The mean time of diagnosis to autoPBHCST was 94 days (range, 48–150 days). Eight dogs were in clinical remission at the time of peripheral blood mononuclear cell apheresis. One dog was in a partial remission at this time and one dog tested B-cell PARR positive (peripheral blood) at this time, but had no other evidence of disease. PARR analysis of all eight harvest products collected from dogs with B-cell PARR positive tumors, including the dog who had B-cell PARR positive blood, was negative (Table 1).

**Table 1:** Patient characteristics before PBHSCT.

**Signalment**	
Number of dogs	10
Breeds	6 (3 Labrador retrievers, 1 great Dane, 1 American cocker spaniel, 1 miniature schnauzer, 1 German shepherd, 1 Pembroke Welsh corgi, 2 mixed breed)
Age (years)	Mean, 5 (range, 3–6)
Spay/neuter	7 NM, 3 SF
Weight (kgs)	Mean, 31 (range, 7.3–66.5)
**B-Cell Lymphoma**	
Stage III	5
Stage IV	2
Stage V	3
Substage a	10
Substage b	0
PARR + tumors	8
Relapse before PBHSCT	2
Disease status at PBHSCT	
Clinical remission	8
PR	2
Time from diagnosis to PBHSCT (days)	Mean, 94 (range, 48–150)
PARR-harvest product	8

*PBHSCT, peripheral blood hematopoietic stem cell transplant; NM, neuteured male; SF, spayed female; PARR, PCR for antigen receptor rearrangements*.

### AutoPBHSCT

For autoPBHSCT, infusing ≥ 2 x 10^6^ CD34+ cells/kg after myeloablation ensures complete hematologic engraftment (1, 2). The median CD34+ percentage of the apheresis products was 1.75% (range, 0.4–4.1%). The median infused CD34+ cell dose was 9.6 x10^6^ cells/kg (range, 2.8 x 10^6^ cells/kg-2.1 x 10^7^ cells/kg). One dog underwent apheresis twice on two consecutive days since the first apheresis did not yield an adequate number of CD34+ cells/kg (1.0 x 10^6^ cells/kg) to proceed. By combining the two harvest products, this dog received an adequate number of infused CD34+ cells (1.2 x 10^7^ CD34+ cells/kg). All dogs engrafted with appropriate hematologic reconstitution kinetics as previously described (21) and were discharged from the hospital ~2 weeks after the transplant.

### Adoptive T-Cell Therapy

After complete hematologic reconstitution post-autoPBHCST, peripheral blood was collected and shipped to a commercial laboratory for CD3+ T-cell expansion at a median of 45.5 days (range, 26–111 days) following autoPBHCST. All dogs were considered in clinical remission at the time of the first infusion. The first ACTs were infused at a median of 74 days (range, 48–132 days) following autoPBHCST. Three (30%) dogs received only one infusion, three dogs (30%) received only two infusions and two dogs received three (20%) and four (20%) infusions, respectively. Seven (70%) dogs received a second infusion a median of 7 days (range, 5–28 days) following the first infusion. One dog received a second T-cell infusion 28 days after the first infusion due to a slower than expected expansion. Four (40%) dogs received a third infusion at a median of 28 days (range, 21–433 days) after the second infusion and two of these dogs also received a fourth infusion seven days later. One dog, who relapsed with disease, received third and fourth T-cell infusions 433 and 440 days after the second T-cell infusion, respectively. The median T-cell concentration of all infusions was 5.62 x 10^6^ cells/kg (range, 2.59 x 10^6^/kg−7.94 x 10^6^ cells/kg). The median T-cell concentration of infusions 1 and 2 (10 dogs) was 8.55 x 10^6^ cells/kg (range, 3.36 x 10^5^-2.58 x 10^7^ cells/kg) and 2.59 x 10^6^ cells/kg (range, 6.96 x 10^5^-1.22 x 10^6^ cells/kg), respectively. All infusions were administered without incident with no immediate or delayed adverse events (AE) observed.

### Outcome

#### Remission Duration

**Figure 1:**
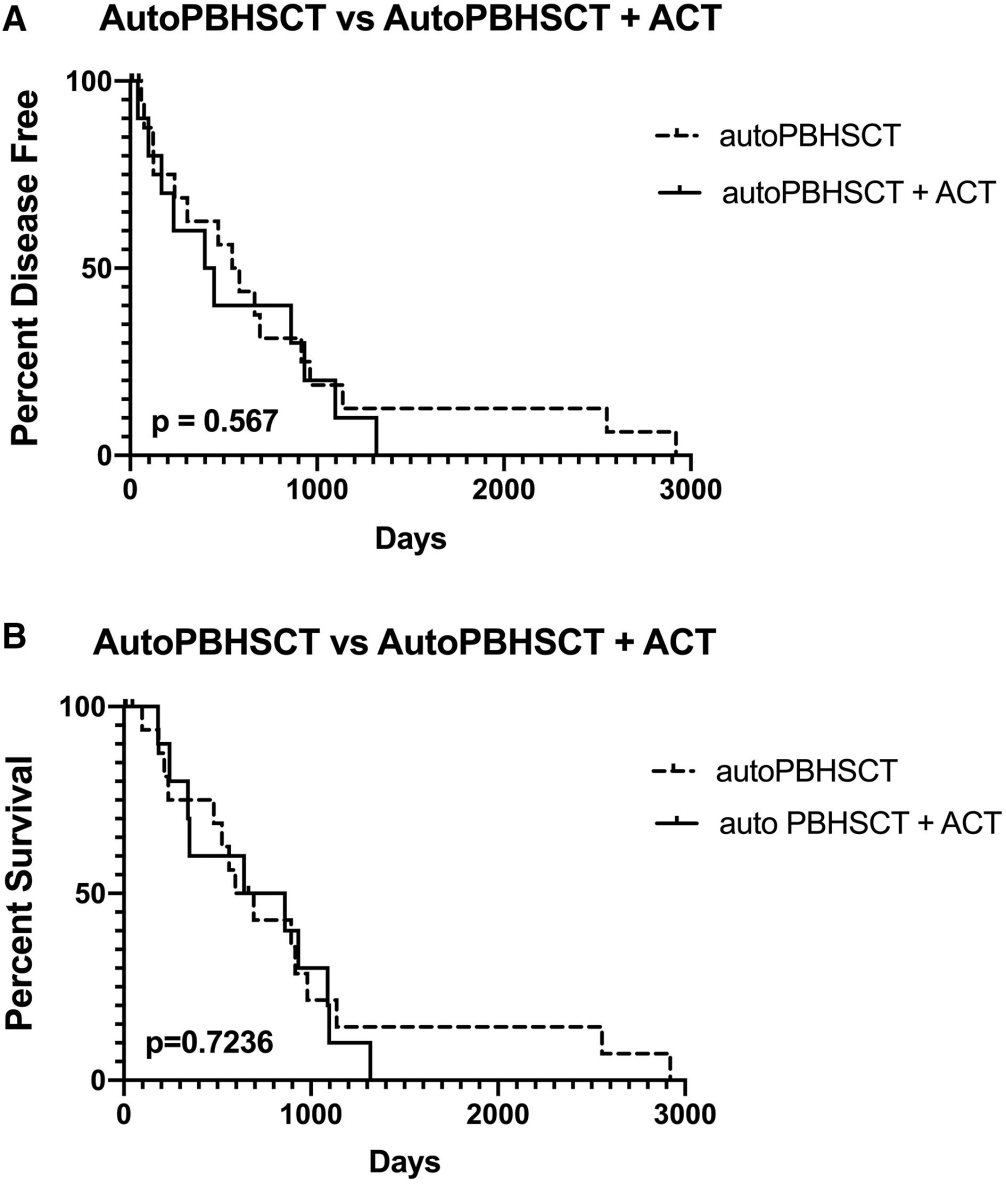
Disease-free interval **(A)** and overall survival **(B)** of 19 dogs treated with autologous peripheral blood hematopoietic cell transplantation (autoPBHCT) alone vs. 10 dogs treated with autoPBHCT and adoptive T-cell therapy (ACT). A *p* value of ≤ 0.05 was considered statistically significant.

**Figure 2:**
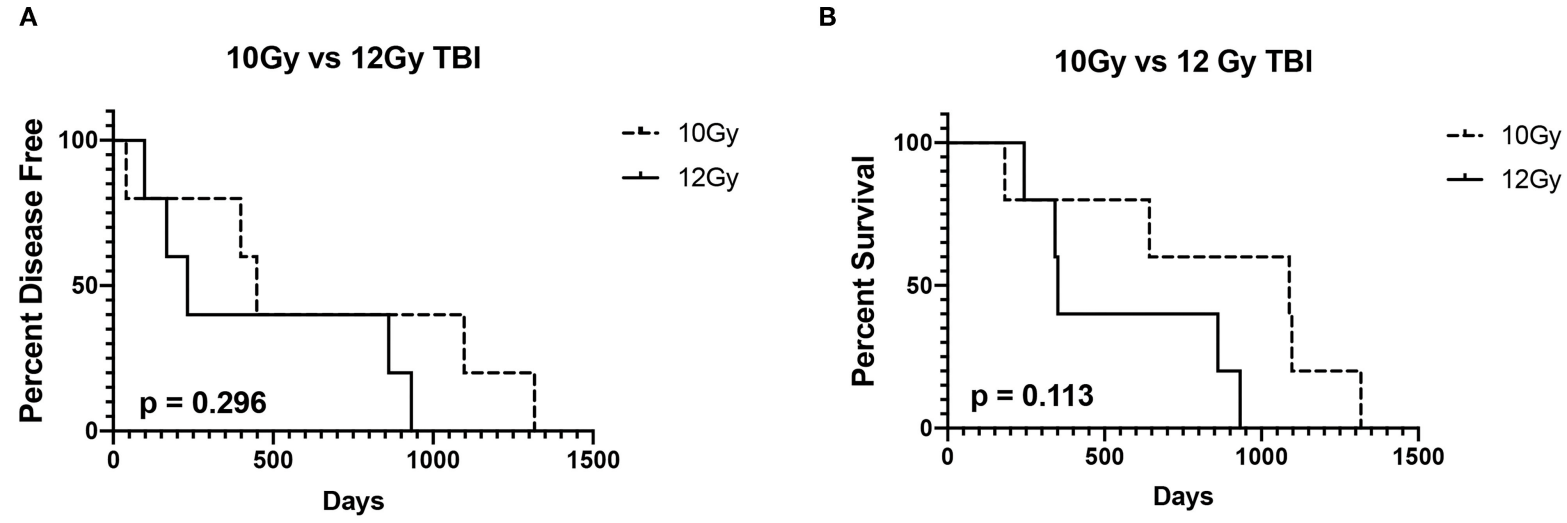
Disease-free interval **(A)** and overall survival **(B)** of 5 dogs treated with 10Gy total body irradiation (TBI) before autologous peripheral blood hematopoietic cell transplantation (autoPBHCT) and adoptive T-cell therapy (ACT) vs. 5 dogs treated with 12GY TBI before autoPBHCST and ACT. A *p* value of < 0.05 was considered statistically significant.

Six (60%) dogs relapsed with disease following autoPBHCST and ACT. Two (20%) dogs relapsed 4 months post-autoPBHSCT, two (20%) dogs relapsed at 5 and 8 months post-autoPBHCST and two (20%) dogs relapsed at 13 and 15 months post-PBHSCT. One dog that was in a partial remission at the time of apheresis had the shortest DFI of 40 days. The median DFI of the dogs from the time of autoPBHCST was 199.5 days (range, 40–449 days). When compared to a historical control group of 15 dogs with B-cell LSA who received CHOP chemotherapy and were in first remission before an autoPBHCST (median DFI = 565 days; range, 59–2920 days) (1), there was no statistically significant difference in DFI (*p* = 0.567) (Figure 1A). In addition, there was no statistically significant difference in DFI between the dogs in this study receiving 10 or 12Gy of TBI (*p* = 0.296) (Figure 2A).

### Overall Survival

Four dogs (40%) are still alive ≥2 years post-autoPBHCST and ACT, in remission at the time of manuscript submission, and are considered cured of their disease, including the dog that tested PARR positive on peripheral blood at the time of apheresis. The median OS of these 4 dogs from the time of autoPBHCST was 1014.5d (range, 86–1317 days). One dog that was in a partial remission at the time of PBHSCT had the shortest OS of 182 days. One dog that relapsed with disease 399 days post-autoPBHSCT lived another 690 days while receiving a variety of chemotherapy protocols leading to an OS of 1,089 days. The median OS of all dogs from the time of autoPBHSCT was 752 days (range, 182–1317 days). When compared to a historical control group of 15 dogs with B-cell LSA who received CHOP chemotherapy and were in first remission before an autoPBHCST (median OS = 531 days; range, 97–2920 days) (1), there was no statistically significant difference in OS (*p* = 0.7236). (Figure 1B). The median OS of all dogs who received autoPBHSCT and ACT from the date of diagnosis was 871 days. In addition, there was no statistically significant difference in OS between the dogs in this study receiving 10 or 12Gy of TBI (*p* = 0.113) (Figure 2B), since two dogs from each cohort were cured of their disease.

### Discussion

This retrospective study describes the feasibility, safety, and efficacy of an aggressive treatment protocol for dogs with high-grade B cell LSA using CHOP chemotherapy, autoPBHSCT, and cellular immunotherapy using ACT. Since our group has previously described the use of CHOP chemotherapy and autoPBHSCT to treat dogs with B-cell LSA, we reasoned that the addition of ACT to the protocol could enhance the clinical benefit of autoPBHCST by eliminating the minimal residual disease that survived TBI responsible for the ~70% relapse rate.

To administer this aggressive treatment protocol, we enlisted a commercial US laboratory to develop a reliable pipeline to perform the technically challenging *in vitro* expansion of CD3+ T-cells, sterilely collect and ship the cells appropriately, and ultimately, have the cells administered locally in a timely manner. Since most dogs who received autoPBHSCT did not live near our facility, effective communication between our institution, the laboratory, and local veterinarians was paramount to ensure the dogs were monitored appropriately after autoPBHCST, blood was collected at the appropriate time, shipments were sent quickly, and multiple doses of expanded T-cells were infused as directed. Given the unpredictability of *in vitro* T-cell expansion, as evidenced by the wide range of days the initial ACT infusion was given after autoPBHSCT (48–132 days), and the total number of doses administered (2–4 doses), the animal caregivers and local veterinarians routinely needed to make scheduling adjustments to expedite the infusion of the product quickly upon arrival (the product was chilled, not frozen). With appropriate communication, the pipeline we developed was reliable enough to make ACT feasible in this setting.

In physician-based medicine, infusion of a variety of *ex vivo* expanded T-cell products (antigen specific cytotoxic T-cells, allo-depleted T-cells, and genetically modified T-cells) can lead to infusion-related AEs. In one large review, the most common AEs were nausea and vomiting (2.62%), likely due to the DMSO cryoprotectant, and hypotension (3.1%) attributable to diphenhydramine pre-medication. (22) Other, less common, AEs included culture negative fever, chills, and nausea only. In the previously cited study (16), Grade I/II diarrhea (2 dogs) and Grade I/II vomiting (2 dogs) and Grades III/IV diarrhea (1 dog) and Grade III/IV vomiting (1 dog) was noted within 72 h after the 2^nd^ of three infusions. These AEs were not noted after the last infusion. However, the dogs were not premedicated with any mediations to mitigate these AEs. In our study, all dogs receiving ACT were given diphenhydramine and maropitant ~15m before each infusion. None of the dogs exhibited any AEs during the first infusion, within an hour of the first infusion, and no dog exhibited any AEs at home after the first infusion. Importantly, we also did not see any AEs during any of the subsequent infusions in any dog, regardless of how many infusions were given. As such, we conclude the ACT product can be given safely over multiple doses with appropriate premedications.

Several hypotheses may explain the lack of efficacy of ACT. Infused adoptive T cells are not resistant to remaining endogenous immune suppression or the immune-suppressive tumor microenvironment. In addition, although the *ex vivo* expansion of T-cells preferentially expands the number of CD8-positive cytotoxic T-cells (16, 23), these cells are not tumor specific, which hinders their ability to break tolerance and engage *in vivo* targets on malignant cells. Importantly, information on the dose and schedule of ACT administration is not standardized in physician-based literature. Evidence suggests that fractionated doses of adoptively transferred T cells are superior to a single infusion (24) and that the ideal dose is related to the tumor burden and the homing characteristics of the infused cells (25). As such, an inaccurate cell dose may prevent full effectiveness. Finally, the ideal time of administration of ACT post-PBHSCT is unknown in both species.

Since this was a small retrospective study, we were not able to compare the outcome with dogs treated using CHOP chemotherapy and autoPBHSCT alone in a prospective fashion. For this reason, we used historical data from a previously published manuscript from our group describing the use of CHOP chemotherapy and PBHSCT alone to treat 15 dogs with B-cell lymphoma in their first clinical remission as historical controls (1). However, despite the promising reported antitumor properties of canine ACT when used to treat only 8 dogs (16), our data does not suggest an overall benefit to the 10 dogs who received CHOP chemotherapy, autoPBHCST and ACT when compared to a historical control group of dogs who received CHOP chemotherapy and autoPBHSCT only. However, there are significant differences between these two studies which makes outcome comparisons difficult, the most important being the phenotype of the ACT product, the cell doses infused, and the variable infusion schedule. In the previous study, the infused product was comprised primarily of CD3+CD8+ T-cells (median CD4:CD8 ratio ~1:9), whereas the infused product used here, based on the data supplied by the company, was composed primarily of CD3+CD4+ T-cells (median CD4:CD8 ratio ~2:1). In addition, dogs were treated with three escalating doses of T-cells [5 x 10^7^ cells/m^2^, 5 x 10^8^/m^2^, and 3 x 10^9^/m^2^ (mean 1.18 x 10^9^ cells/m^2^)] administered 2 weeks apart in the previous study, while dogs in this study were treated with from 1 to 4 infusions of much low cell doses (infusion #1 mean 2.6 x 10^8^/m^2^) in a much less structured schedule. The highest single cell dose given to a dog in this study was 7.6 x 10^8^ cells/m^2^. For these, and other reasons [i.e., small sample size in both studies, when the peripheral blood cells were collected for *in vitro* expansion, how the final ACT product was delivered (cryopreserved vs. chilled)], comparison of outcomes cannot be confidently ascertained.

### Conclusions

To the author's knowledge, this is the first report describing an aggressive treatment option for dogs with B-cell lymphoma that combines CHOP chemotherapy, autoPBHCST, and ACT. Based on this small retrospective study, we can only suggest that ACT infusions after CHOP chemotherapy and autoPBHCST for the treatment of dogs with B-cell lymphoma does not provide a clinical benefit over CHOP chemotherapy and autoPBHSCT alone. Although 40% of dogs in this study have lived >2 years post-autoPBHCST and are considered cured of their disease compared to 30% in our previous study (1), the sample size was not sufficient to determine if the increased cure rate was due to the addition of ACT to our autoPBHSCT protocol. Importantly, however, this study does corroborate our previous findings that a population of dogs diagnosed with B-cell lymphoma can be cured using a combination of CHOP chemotherapy and autoPBHCT (1). Additional, larger studies in dogs are needed to answer challenging unanswered questions relating to the timing of peripheral blood collection and T-cell expansion, the optimal *in vitro* ACT expansion protocol and infusion cell dose, and optimal infusion schedules in the setting of autoPBHSCT.

## Data Availability Statement

The original contributions presented in the study are included in the article/Supplementary Materials, further inquiries can be directed to the corresponding authors.

## Author Contributions

All authors contributed equally to the collection of data, analysis of data, and writing this manuscript.

## Conflict of Interest

The authors declare that the research was conducted in the absence of any commercial or financial relationships that could be construed as a potential conflict of interest.

## Publisher's Note

All claims expressed in this article are solely those of the authors and do not necessarily represent those of their affiliated organizations, or those of the publisher, the editors and the reviewers. Any product that may be evaluated in this article, or claim that may be made by its manufacturer, is not guaranteed or endorsed by the publisher.

## Supplementary Material

Supplementary Figure 1 Flow cytometric analysis of final *in vivo* expanded peripheral blood T-cell product using flow cytometry (Hematologics, Inc) and standard antibodies against canine blood cell antigens. After cell size and granularity analysis **(A)**, the cells were stained with 7-AAD to identify viable cells **(B)**. The viable cells were identified as viable CD45+ leukocytes **(C)** before determining the number of CD21+ B-cells and CD5+ T-cells **(D)**. Finally, CD5+CD4+ and CD5+CD8+ subsets were quantitated **(E)**.

Supplementary Figure 2 A custom manufactured BioLoc^TM^ needle-free cell transfer bag (Instant Systems, Inc., Norfolk, VA, US) was used to ship the final chilled T-cell product.

Supplementary Figure 3 Package insert included with the final shipped ACT product generated by Aurelius Biotherapeutics^©^.

Supplementary Table 1 Flow cytometric analysis of 16 *in vitro* expanded T-cell products produced by Aurelius Biotherapeutics^©^.
