# Supplementary figures and images for: A Retrospective Analysis: Autologous Peripheral Blood Hematopoietic Stem Cell Transplant Combined With Adoptive T-Cell Therapy for the Treatment of High-Grade B-Cell Lymphoma in Ten Dogs
Source: Front Vet Sci. 2021 Dec 7;8:787373. doi: 10.3389/fvets.2021.787373 (PMC8688351; doi:10.3389/fvets.2021.787373)

S1

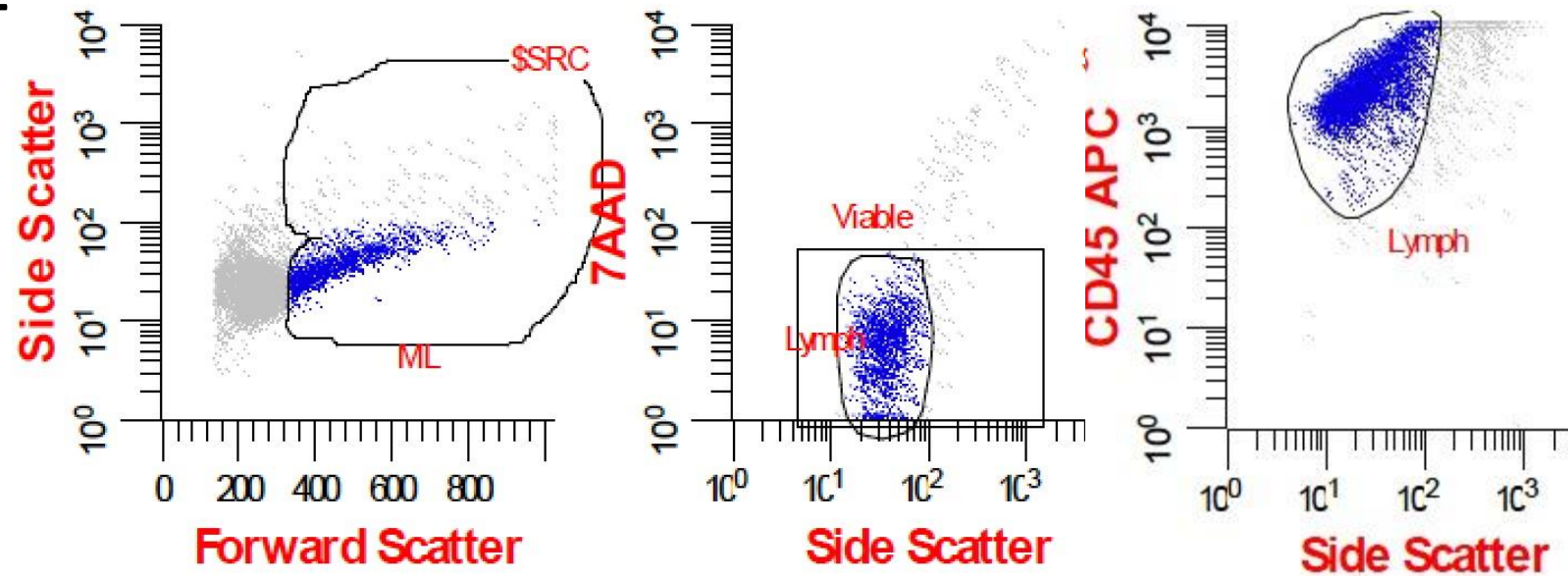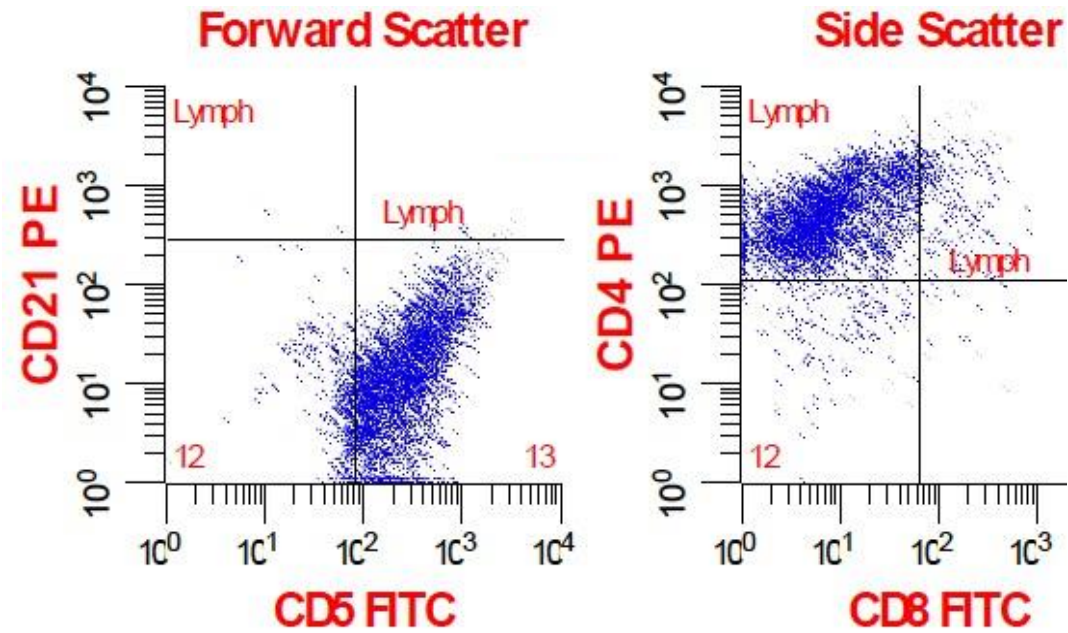

Supplement: Supplementary Figure 1 — Flow cytometric analysis of final in vivo expanded peripheral blood T-cell product using flow cytometry (Hematologics, Inc) and standard antibodies against canine blood cell antigens. After cell size and granularity analysis (A), the cells were stained with 7-AAD to identify viable cells (B). The viable cells were identified as viable CD45+ leukocytes (C) before determining the number of CD21+ B-cells and CD5+ T-cells (D). Finally, CD5+CD4+ and CD5+CD8+ subsets were quantitated (E). [file Image_1.pdf]

S2

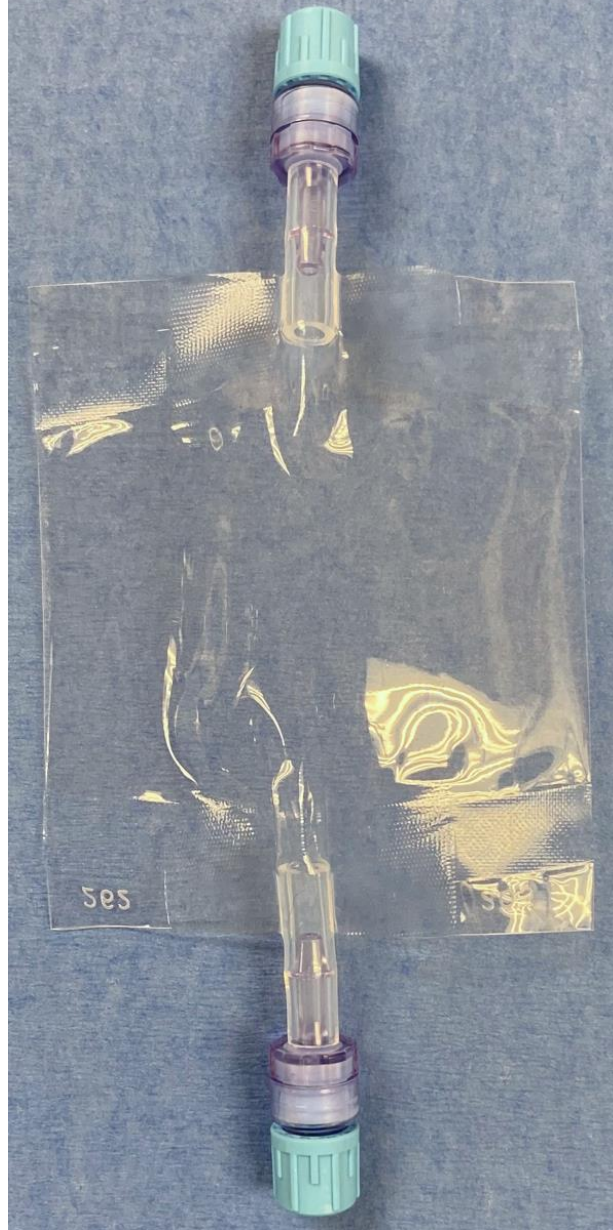

Supplement: Supplementary Figure 2 — A custom manufactured BioLocTM needle-free cell transfer bag (Instant Systems, Inc., Norfolk, VA, US) was used to ship the final chilled T-cell product. [file Image_2.pdf]
