# Supplementary material for: A Retrospective Analysis: Autologous Peripheral Blood Hematopoietic Stem Cell Transplant Combined With Adoptive T-Cell Therapy for the Treatment of High-Grade B-Cell Lymphoma in Ten Dogs
Source: Front Vet Sci. 2021 Dec 7;8:787373. doi: 10.3389/fvets.2021.787373 (PMC8688351; doi:10.3389/fvets.2021.787373)
Supplement: Supplementary Figure 3 — Package insert included with the final shipped ACT product generated by Aurelius Biotherapeutics©. [file Image_3.pdf]

S3

Package Insert for Autologous Prescription Product - Activated Autologous T cells

Description: Autologous cancer antigen-specific T cell infusion.

I**ndications**: For the treatment of canine B cell lymphoma.

Dosage and administration: This product has been created from autologous patient T cells that have been expanded and activated. Infuse intravenously by personnel experienced with transfusion procedures. Aseptically place a 22 or 20 gauge intravenous catheter into a peripheral vein. Just prior to infusion, the bag should be gently rocked to ensure that the cells are suspended in solution. Remove the cap from a needle free port on the bag, swab the port with 70% isopropyl alcohol, attach a sterile 20 ml syringe (included)and gently aspirate the entire volume of the bag into the syringe. Attach the provided T port to the catheter, and attach the syringe to the T port, slowly inject the infusion over a 2-3 minute period. Instill 20 ml of sterile, room temperature 0.9% NaCl into the T cell bag to rinse the remainder of the cells out of the bag and aspirate with syringe and inject the final volume through the T port into the catheter.

Contraindication: Must be administered intravenously to the patient identified on the label. In case of human exposure, contact a physician.

Precautions: Before administration confirm that the patient ID and accession number match the number on the T cell infusion bag. Restricted to use by a veterinarian.

Warning: Keep this and all medications out of reach of children. Not for use in humans. Inactivate unused contents before disposal.

Included materials: BioLoc ™cell transport bag, 20 ml activated T cell product, sterile needle free T port, infusion protocol sheet, 20 ml sterile syringe, alcohol swab, adverse reaction sheet and patient identification sheet.

Adverse reactions: Nausea, vomiting, diarrhea, fever, are uncommon, but may occur during or shortly after infusion. If so, decrease infusion rate to 1 ml/min. Contact Aurelius Biotherapeutics if adverse reactions are observed.

Storage information: The T cell infusion product should be refrigerated (2 - 6℃ to 35-46℉) upon arrival. Allow approximately 5 minutes for the product to warm to room temperature prior to the infusion. The product must be used within 4 hours of reaching room temperature.

How supplied: The T cell product is supplied in a 40 ml Bioloc™needle free cell transport bag containing approximately 20 ml of opaque, slightly pink solution that has a minimum concentration of 1×10^5 activated T lymphocytes/kg body weight.

Manufactured by:
Aurelius Biotherapeutics 720 Virginia Street Bellingham, WA 98225

Prod. code no.75A7.50 U.S. Permit No. For Experimental use only. Not for sale.

Aurelius Bio info@aureliusbio.com 360.734.0720 ext #106 720 Virginia Street Bellingham WA 98225
