# Supplementary material for: A Retrospective Analysis: Autologous Peripheral Blood Hematopoietic Stem Cell Transplant Combined With Adoptive T-Cell Therapy for the Treatment of High-Grade B-Cell Lymphoma in Ten Dogs
Source: Front Vet Sci. 2021 Dec 7;8:787373. doi: 10.3389/fvets.2021.787373 (PMC8688351; doi:10.3389/fvets.2021.787373)
Supplement: Supplementary Table 1 — Flow cytometric analysis of 16 in vitro expanded T-cell products produced by Aurelius Biotherapeutics©. [file Table_1.pdf]

Sample	CD45- (%)	CD45+ (%)	CD45- NK cells (%)	CD45+/CD3+ lymphocytes (%)	CD4/CD8 ratio
1	77	19	1.5	98.5	1:1
2	82	18	21	79	1.5:1
3	88	12	-	100	7.5:1
4	42	58	-	100	1:0
5	95	4.8	-	100	8.9:1
6	48	52	5.4	94.6	0.03:1
7	8	92	-	100	2:1
8	85	15	-	100	4.7:1
9	7	93	-	100	2:1
10	83	82	-	100	3.7:1
11	43	57	-	100	1:1
12	41	59	21	79	1.9:1
13	84	16	-	100	1:0
14	55	45	-	100	29:1
15	23	77	-	100	49:1
16	59	41	-	100	5.6:1
**Median**	**57**	**53**	**-**	**100**	**2:1**
